# Social Convoys Across the Lifespan and Links With Later-Life Well-Being and Societal Trust

**DOI:** 10.1093/geroni/igaf122.1860

**Published:** 2025-12-31

**Authors:** Rita Hu, Noah Webster, Elaine Wethington

## Abstract

Social relationships evolve across the lifespan and have great potential to shape individual and societal outcomes. This symposium examines how early-life bonds, caregiving experiences, and social network characteristics influence aging trajectories and their broader implications for macro-level societal trust. Drawing on multiple population-level and longitudinal studies, this set of papers together highlight recent innovations in social convoy research. Garcia and Antonucci examine how childhood closeness to grandparents predicts midlife depressive symptoms, stress and self-efficacy. Their findings demonstrate the lasting emotional benefits of intergenerational relationships. Hu and Lee investigate earlier-life caregiving histories and their long-lasting influence on later-life social disconnectedness and loneliness. Results suggest caregiving is linked to greater social participation and less loneliness, particularly among men, underscoring positive effects on caregivers’ later-life social well-being. Iveniuk and Ricketts examine how social network turnover affects mental health. Their findings reveal race and gender differences in patterns of network turnover. They also found that the loss of weaker ties is linked with more depressive symptoms, while gaining new close confidants is associated with fewer depressive symptoms. Their study provides new insights into how network stability and change influence mental health disparities. Webster and colleagues explore how social network structure and composition shape macro-level personal trust. They found that larger inner circles and more non-family ties foster societal trust, linking interpersonal relationships to broader social cohesion. Discussant Elaine Wethington, a leading expert in social relationships and translational research, will synthesize these findings and discuss implications for interventions and policies that foster lifelong social convoys.

